# Inhibition of Chk1 with Prexasertib Enhances the Anticancer Activity of Ciclopirox in Non-Small Cell Lung Cancer Cells

**DOI:** 10.3390/cells13211752

**Published:** 2024-10-23

**Authors:** Zhu Huang, Wenjing Li, Yan Wu, Bing Cheng, Shile Huang

**Affiliations:** 1 Department of Biochemistry and Molecular Biology, Louisiana State University Health Sciences Center, Shreveport, LA 71103, USA; huangzhu@xmu.edu.cn (Z.H.);; 2 Collaborative Innovation Center of Targeted Development of Medicinal Resources, College of Life Science, Anqing Normal University, Anqing 246011, Anhui, China; zhuliangliang@aqnu.edu.cn (W.L.);; 3 Department of Hematology and Oncology, Louisiana State University Health Sciences Center, Shreveport, LA 71103, USA; 4 Feist-Weiller Cancer Center, Louisiana State University Health Sciences Center, Shreveport, LA 71103, USA

**Keywords:** ciclopirox, prexasertib, Chk1, cell cycle, apoptosis, non-small cell lung cancer

## Abstract

Lung cancer is a leading cause of cancer-related deaths worldwide. Non-small cell lung cancer (NSCLC) is the most prevalent lung cancer subtype. Ciclopirox olamine (CPX), an off-patent fungicide, has been identified as a new anticancer agent. Prexasertib (PRE), a Chk1 inhibitor, is in phase 1/2 clinical trials in various tumors. The anticancer effect of the combination of CPX with PRE on NSCLC cells is unknown. Here, we show that CPX is synergistic with PRE in inhibiting cell proliferation and inducing apoptosis of NSCLC (A549 and A427) cells. Combined treatment with CPX and PRE significantly increased the cell population in the G1/G0 and sub-G1 phases, compared to the single treatment with CPX or PRE. Concurrently, the combined treatment downregulated the protein levels of cyclins (A, B1), cyclin-dependent kinases 4, 6, 2 (CDK4, CDK6, CDK2), cell division cycle 25 B, C (Cdc25B, Cdc25C), and upregulated the protein levels of the CDK inhibitors p21 and p27, leading to decreased phosphorylation of Rb. In addition, the combined treatment increased DNA damage, evidenced by increased expression of γH2AX. In line with this, the combined treatment induced more apoptosis than either single treatment. This was associated with increased expression of DR4, DR5, Fas, and FADD and decreased expression of survivin, resulting in activation of caspase 8 and caspase 3 as well as cleavage of poly (ADP ribose) polymerase (PARP). Taken together, the results suggest that inhibition of Chk1 with PRE can enhance the anticancer activity of CPX at least partly by decreasing cell proliferation and increasing apoptosis in NSCLC cells.

## 1. Introduction

Lung cancer, including small cell lung cancer and non-small cell lung cancer (NSCLC), is the leading cause of cancer-related deaths worldwide [1]. Approximately 10–15% of all lung cancers are small cell lung cancer, and about 80–85% are NSCLC [1]. Unfortunately, the 5-year survival rate of lung cancer remains less than 20% globally despite application of targeted therapies and immunotherapies [2,3]. Patients with NSCLC are commonly diagnosed with advanced disease and have limited therapeutic options. Currently, chemotherapy remains the mainstay for most of NSCLC patients [4]. However, drug resistance and tumor recurrence occur frequently [4]. Additionally, the severe side effects of chemotherapy are inevitable and sometimes intolerable [4]. Thus, there is an urgent need to identify novel effective therapies for the aggressive disease.

Ciclopirox olamine (CPX), an off-patent fungicide, is widely used to treat fungal infection of the skin and nails [5,6]. Apart from its broad-spectrum antifungal and antibacterial activity, CPX also shows anticancer activity against a spectrum of human tumors, which is related to its inhibition of cell proliferation, induction of apoptosis, suppression of cell migration and invasion, and inhibition of angiogenesis and lymphangiogenesis [7]. The molecular mechanisms of CPX anticancer action are multiple and complex. CPX alters the expression or activities of multiple signaling molecules or pathways, such as ribonucleotide reductase (RR) [8,9], cyclin dependent kinases (CDKs) [10,11], Myc [12], DJ-1 [13], Wnt/β-catenin [14,15], DOHH/eIF5A/PEAK1 [16,17,18], VEGFR-3/ERK1/2 [19], ATR/Chk1/Cdc25A [20,21], and AMPK/TSC/mTORC1 [22,23,24]. Most of these effects are attributable to the iron chelation activity of CPX [7]. Our previous study has demonstrated that CPX treatment induces DNA damage and activates the checkpoint kinase 1 (Chk1) pathway, thereby preventing damaged cells from progressing through the cell cycle [21]. Since the cell cycle arrest contributes to decreased cell proliferation, but it also facilitates DNA damage repair [25], we hypothesized that inhibition of Chk1 may enhance CPX-induced cell death.

Chk1, belonging to the cell cycle check point protein, is phosphorylated and activated by ATM/ATR (ataxia telangiectasia mutated/ataxia telangiectasia and rad3-related protein) in response to DNA replication stress or DNA damage [26,27]. Overexpression of Chk1 has been documented in numerous human tumors, which contributes to resistance to chemotherapy or radiotherapy [28]. Thus, Chk1 has been recognized as a promising target for cancer therapy. Prexasertib (PRE), also named LY2606368, is a small ATP-competitive inhibitor mainly against Chk1 and minorly against Chk2, which has shown promising anticancer activity in preclinical studies [29]. PRE, in single or combination treatments with diverse chemotherapeutic agents, is in phase I and phase II clinical trials for multiple types of tumors [29]. It has been shown that PRE treatment can result in DNA double strand breaks (DSB) and cell death in the cells of S-phase presumptively by causing replication catastrophe [30,31,32,33]. So far, whether PRE can enhance the anticancer activity of CPX in NSCLC has not been explored.

Here, we show that the combination of CPX and PRE exhibits a synergistic anticancer activity in NSCLC cells in vitro. Mechanistically, PRE potentiates CPX-induced DNA damage, cell proliferation inhibition and apoptosis in the tumor cells. Our results suggest that the CPX/PRE combination is a new promising therapeutic approach for treatment of NSCLC.

## 2. Materials and Methods

### 2.1. Materials

CPX (Sigma, St. Louis, MO, USA) was prepared to 100 mM stock solution by dissolving it in 100% ethanol and stored at −20 °C. PRE (Selleckchem, Houston, TX, USA) was prepared to 10 mM stock solution by dissolving it in dimethyl sulfoxide (DMSO) and stored at −20 °C. Fetal bovine serum (FBS) was purchased from Biotechne (Minneapolis, MN, USA). Dulbecco’s Modified Eagle Medium (DMEM) and 0.05% Trypsin-EDTA were obtained from Mediatech (Herndon, VA, USA). 5-bromo-2-deoxyuridine (BrdU) and 4′,6-diamidino-2-phenylindole (DAPI) were from Life Technologies (Grand Island, NY, USA). The primary antibodies included antibodies to γH2AX, cyclin D1, cyclin E, cyclin A, cyclin B1, CDK4, CDK6, CDK2, CDK1, Cdc25A, Cdc25B, Cdc25C, p27^Kip1^, p21^Cip1^, Rb, p-Rb (S807/811), p-Rb (S780), Bcl-2, Bcl-xL, Mcl-1, survivin, BAD, BAK, BAX, RIP, FasL, Fas/CD95, TRADD, FADD, TRAIL, TNFα, TNFR1, DR4, DR5, BrdU and β-actin (Santa Cruz Biotechnology, Dallas, TX, USA), cleaved PARP, cleaved caspase 3, cleaved caspase 8, and cleaved caspase 9 (Cell Signaling, Danvers, MA, USA). Goat anti-rabbit IgG-horseradish peroxidase (HRP) and goat anti-mouse IgG-HRP were purchased from Pierce (Rockland, IL, USA). Goat anti-mouse IgG1-Alexa Fluor 488 and goat anti mouse IgG1-Alexa Fluor 594 were purchased from Invitrogen (Carlsbad, CA, USA). All other chemicals were purchased from Sigma (St. Louis, MO, USA), unless otherwise stated.

### 2.2. Cell Lines and Cultures

Human NSCLC (A549 and A427) cell lines (American Type Culture Collection, Manassas, VA, USA) were grown in DMEM containing 10% FBS, and cultured in a humidified incubator (37 °C and 5% CO_2_). The authentication of each cell line used was not confirmed in this study. In all treatments, the subconfluent cells (around 70% confluence) were treated with indicated concentrations of CPX or PRE alone or in combination in the growth medium. Cells treated with vehicle (ethanol and/or DMSO, final concentration in the medium = 0.1%) served as a control.

### 2.3. Cell Viability and Proliferation Assays

Cells, seeded in 96-well plates (at a density of 4000–6000 cells per well for cell viability assay) or in 6-well plates (at a density of 5 × 10^4^ cells per well for cell proliferation assay) in triplicates, were grown under a standard culture condition. The next day, the cells were treated with CPX (0, 0.625, 1.25, 2.5, 5 and 10 μM) with or without PRE (2.5, 5, 10, and 20 nM). Treatment with vehicle served as a control. In 72 h or 5 days, cell viability or cell proliferation was evaluated by the MTS assay (Promega, Madison, WI, USA) or cell counting, as described previously [10]. Half-maximal inhibitory concentrations (IC_50_) values were calculated using dose–response curves with the Prism software (Version 9.5) (GraphPad Software, San Diego, CA, USA). All experiments were repeated at least 3 times.

### 2.4. Drug Combination Analysis

Drug combination was analyzed by the Chou–Talalay method [34], which accounts for both the potency (median inhibitory concentration) and the shape of the dose–effect curve. The cytotoxicity of the drug combinations was compared with that of each drug alone using the combination index (CI), with CI values <1, =1 and >1 indicating synergism, additive effect, and antagonism, respectively. The CI was determined using the CompuSyn software (Version 1.0) (https://www.combosyn.com/) accessed on 20 October 2019.

### 2.5. Cell Cycle Analysis

The cell cycle profile was analyzed as described [10]. Briefly, cells, plated in 100 mm dishes (5 × 10^5^ cells per dish), were cultured under a standard culture condition. The next day, after pre-incubation with or without PRE (20 nM) for 2 h, the cells were treated with CPX (0, 5 μM) for 24 h. Then, the cells were trypsinized, washed with cold phosphate-buffered saline (PBS), and stained with the Cellular DNA Flow Cytometric Analysis Kit (Roche Diagnostics, Indianapolis, IN) following the supplier’s instruction. Percentages of cells in each phase (G1/G0, S, or G2/M) of the cell cycle were determined with a FACSCalibur flow cytometer (Becton Dickinson, San Jose, CA, USA) and ModFit LT analyzing software (Version 4.0) (Verity Software House, Topsham, ME, USA). Cells treated with vehicle alone were used as a control.

### 2.6. Apoptosis Assay

Apoptosis was determined as described [10]. Briefly, cells, plated in 100 mm plates (2 × 10^5^ cells per well), were grown under a standard culture condition. The next day, after pretreatment with or without prexasertib (20 nM) for 2 h, the cells were exposed to CPX (0, 5 μM) for 72 h. The cells were then trypsinized, washed with cold PBS, and stained with the Annexin V-FITC Apoptosis Detection Kit I (BD Biosciences, San Jose, CA, USA), followed by flow cytometry on a FACSCalibur flow cytometer (Becton Dickinson). The percentages of cells undergoing apoptosis were analyzed using FACS Diva software (Version 8.0) (BD Bioscience, San Jose, CA, USA). Cells treated with vehicle alone were used as a control.

### 2.7. Western Blot Analysis

Cells, seeded in 6-well plates (6 × 10^5^ cells per well), were grown under a standard culture condition. The next day, after pretreatment with or without prexasertib (20 nM) for 2 h, the cells were treated with CPX (0–20 μM) for 24 h. Subsequently, whole cell lysates were prepared, followed by Western blotting as described [10]. Finally, the bands for individual proteins were semi-quantified using NIH ImageJ software (Version 1.51) (National Institutes of Health, Bethesda, MD, USA).

### 2.8. BrdU Labeling and Staining

Cells, seeded on coverslips in 6-well plates (2 × 10^5^ cells per well), were grown overnight. After pretreatment with or without PRE (20 nM) for 2 h, the cells were treated with CPX (0 and 5 μM) for 24 or 48 h, followed by BrdU labeling and staining, as described [20].

### 2.9. Immunofluorescence Staining for γH2AX

Following treatments, cells were briefly washed with cold PBS, fixed with 4% paraformaldehyde for 1 h at room temperature, and permeabilized with 0.2% Triton X-100 for 5 min. After 1 h blocking with 5% normal goat serum (diluted in PBS containing 0.1% Triton X-100), the cells were incubated with mouse anti-γH2AX antibody (1:200, sc-517348) for 2 h at room temperature and incubated with goat anti-mouse IgG1-Alexa Fluor 594 for 1 h at room temperature in the dark. Subsequently, slides were mounted, fluorescent images were acquired, and the number of γH2AX positive cells were quantified as described above.

### 2.10. Statistical Analysis

All results were presented as mean values ± standard deviation (mean ± SD). Data were analyzed using Prism software (Version 9.5) (GraphPad Software, San Diego, CA, USA). Group variability and interaction were compared using Student’s *t*-test or one-way ANOVA followed by Bonferroni’s post-tests to compare replicate means. A level of *p* < 0.05 was considered statistically significant.

## 3. Results

### 3.1. CPX Is Synergistic with PRE in Reducing Cell Viability and Inhibiting Cell Proliferation of NSCLC Cells

**Figure 1:**
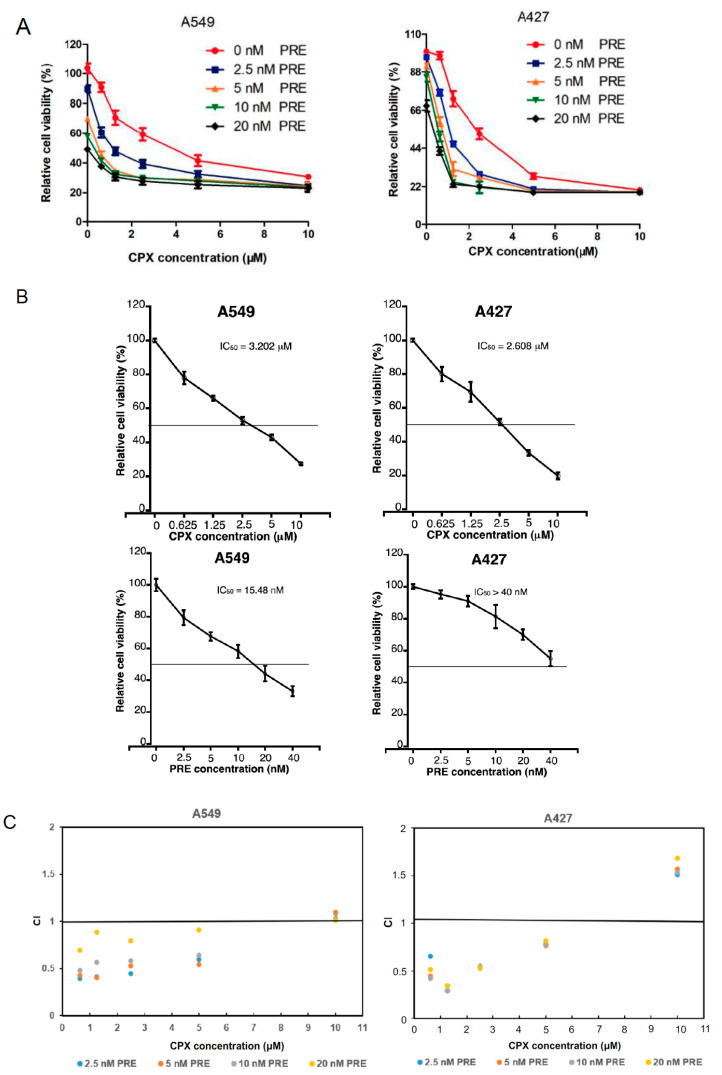
CPX is synergistic with PRE in reducing NSCLC cell viability. (**A**) A549 and A427 cells were treated with CPX (0–10 μM) in the presence or absence of PRE (0–20 nM) for 72 h, followed by MTS assay. Results are presented as mean ± SD (*n* = 6). (**B**) The IC_50_ values of CPX and PRE are shown for indicated cells. (**C**) The combination index (CI) values for CPX and PRE are shown for indicated cells. CI = 1, <1, or >1 indicates additive, synergistic, or antagonistic effect.

To determine the anticancer potential of the combined treatment with CPX and PRE in NSCLC cells, firstly we tested if PRE enhances the cytotoxicity of CPX in NSCLC cells. For this, A549 and A427 cells were treated with CPX (0–10 μM) with or without PRE (0–20 nM) for 72 h, followed by the MTS assay. The results showed that treatment with CPX or PRE alone reduced cell viability in a dose-dependent manner (Figure 1A). The IC_50_ values of CPX were approximately 3.2 and 2.6 μM for A549 and A427 cells, respectively; and the IC_50_ values of PRE were about 15.48 and >40 nM for the two cell lines, respectively (Figure 1B). The addition of PRE strengthened the cytotoxic effect of CPX on the cells (Figure 1A). Analyzed by the Chou–Talalay method [34], when CPX concentrations were ≤5 μM, the combination index (CI) values of CPX/PRE were <1.0, indicating synergism (Figure 1C). However, when CPX concentrations were ≥10 μM, the CI values were >1.0, indicating antagonism (Figure 1C). The results indicate that PRE can synergistically potentiate the cytotoxicity of low concentrations (≤5 μM) of CPX in the NSCLC cells.

**Figure 2:**
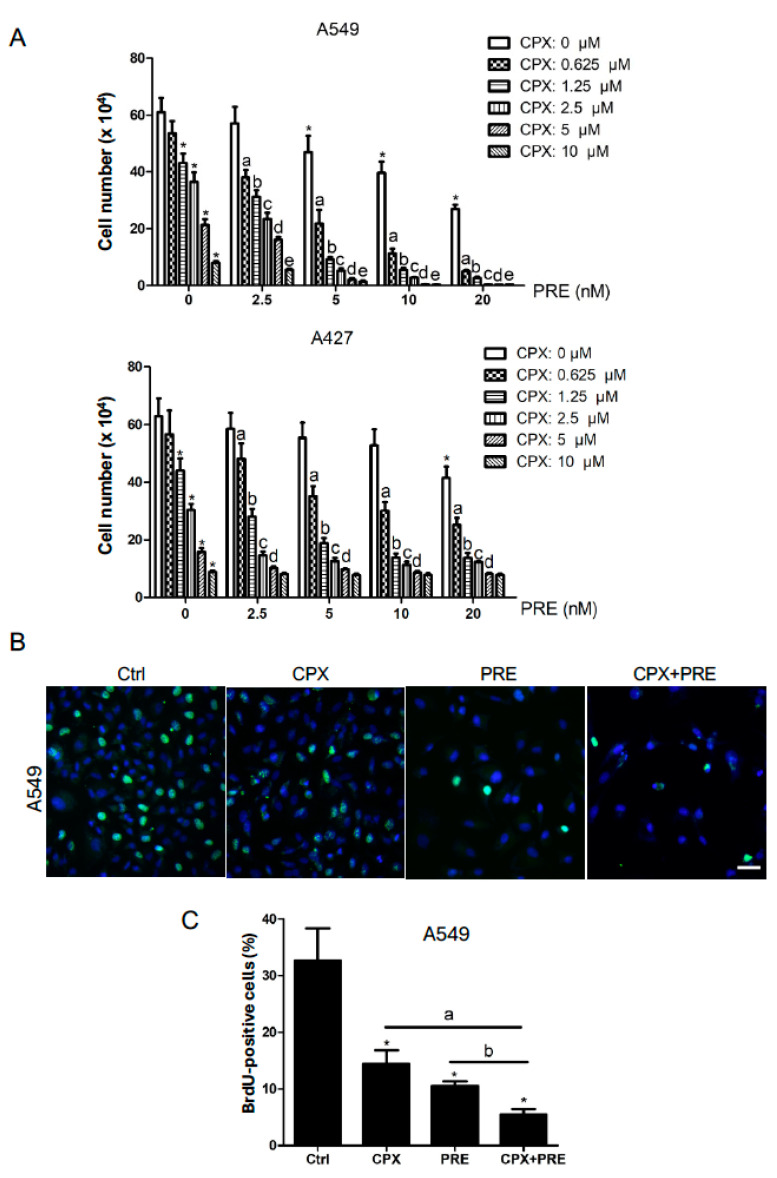
PRE enhances the anti-proliferative effect of CPX on NSCLC cells. (**A**) A549 and A427 cells were treated with CPX (0–10 μM) in the presence or absence of PRE (0–20 nM) for 5 days, followed by cell counting. Results are presented as mean ± SD (*n* = 6). *: *p* < 0.05, compared to the control group (vehicle treatment group); a, b, c, d, and e: *p* < 0.05, compared to the corresponding concentration of CPX alone treatment group. (**B**,**C**) A549 cells were treated with CPX (5 μM) or PRE (20 nM) alone or in combination for 48 h, and then pulsed with BrdU (3 μg/mL) for 3 h, followed by BrdU immunofluorescence staining (green) and DAPI staining (blue). Representative images are shown in (**B**) (Bar = 20 μm), and quantitative analysis of BrdU-positive cells is shown in (**C**). Results are presented as mean ± SD (*n* = 3). *: *p* < 0.05, compared to the control group (vehicle treatment group); a: *p* < 0.05, CPX treatment group vs. CPX + PRE treatment group; and b: *p* < 0.05, PRE treatment group vs. CPX + PRE treatment group.

Next, we examined the anti-proliferative effect of CPX and PRE on the NSCLC cells. For this, A549 and A427 cells were exposed to CPX (0–10 μM) with or without PRE (2.5–20 nM) for 5 days. By cell counting, we found that treatment with CPX alone dose-dependently reduced the cell number of A549 or A427 cells; the addition of PRE significantly enhanced the anti-proliferative effect of CPX (Figure 2A). To substantiate this finding, A549 cells were treated with CPX (5 μM) or PRE (20 nM) alone or in combination for 48 h, and then pulsed with BrdU for 3 h, followed by BrdU immunofluorescence staining. As shown in Figure 2B,C, treatment with CPX or PRE alone for 48 h significantly reduced the percentage of BrdU-positive cells. Of note, the combined treatment with CPX and PRE for 48 h exhibited a more inhibitive effect on BrdU labeling in the cells, indicating inhibition of DNA synthesis or cell proliferation. The results suggest that inhibition of Chk1 with PRE also potentiates the anti-proliferative effect of CPX on NSCLC cells.

### 3.2. Combined Treatment with CPX and PRE Induces Excessive DNA Damage in NSCLC Cells

**Figure 3:**
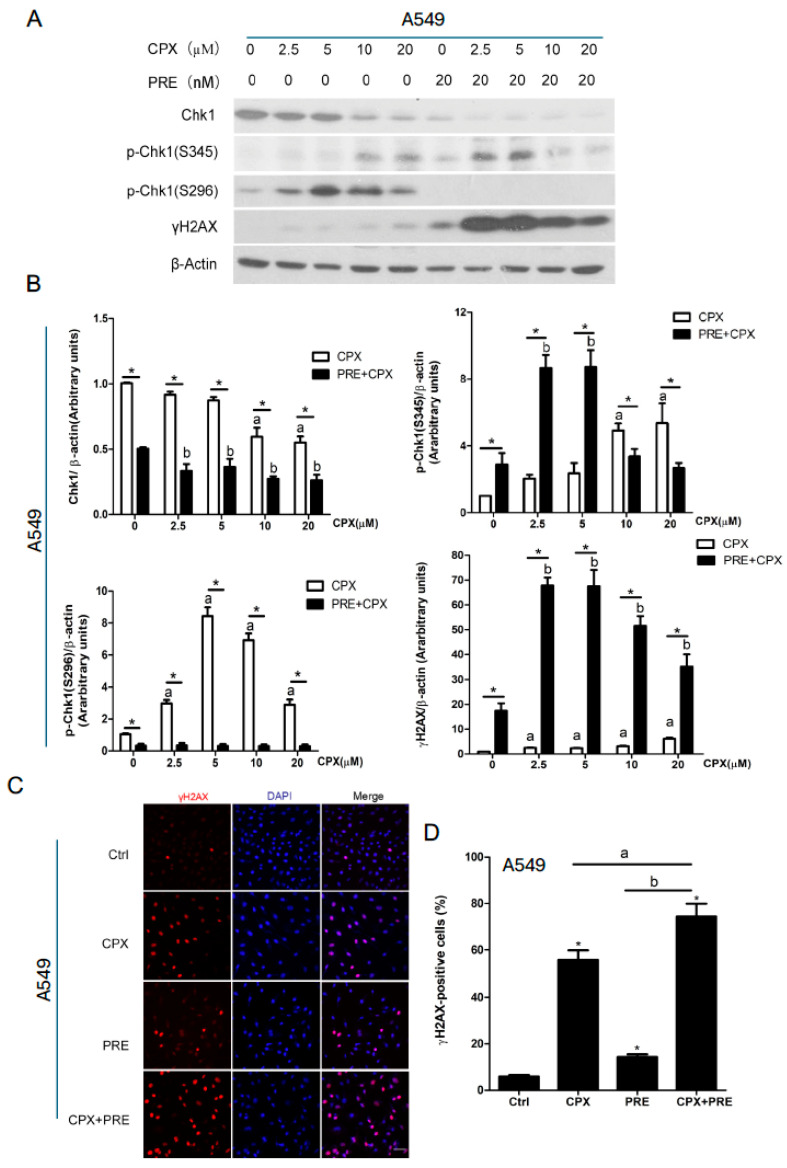
Inhibition of Chk1 with PRE strengthens CPX-induced DNA damage in NSCLC cells. (**A**–**D**) A549 cells were pretreated with or without PRE (20 nM) for 2 h and then exposed to CPX (0–20 μM) for 24 h (for Western blotting) or 48 h (for γH2AX immunofluorescence staining). (**A**) The whole cell lysates were subjected to Western blotting with the indicated antibodies. β-actin served as a loading control. Similar results were obtained in at least three independent experiments. (**B**) The protein bands in the Western blots were semi-quantified using NIH ImageJ. Results are presented as mean ± SD (*n* = 6). * *p* < 0.05, CPX treatment group vs. CPX + PRE treatment group; a: *p* < 0.05, Control vs. CPX treatment group; and b: *p* < 0.05, Control vs. PRE treatment group. (**C**,**D**) γH2AX (red) in A549 cells was detected by immunofluorescence staining. Cell nuclei were counterstained with DAPI (blue). Representative images are shown in (**C**) (Bar = 20 μm), and quantitative analysis of γH2AX-positive cells is shown in (**D**). Results are presented as mean ± SD (*n* = 3). * *p* < 0.05, compared to control group; a: *p* < 0.05, CPX treatment group vs. CPX + PRE treatment group; and b: *p* < 0.05, PRE treatment group vs. CPX + PRE treatment group.

It has been shown that CPX can induce DNA damage and activate the ATR-Chk1 pathway in breast cancer (MDA-MB-231) and rhabdomyosarcoma (Rh30) cells [21]. Next, we validated whether CPX has this effect on NSCLC cells. As expected, treatment with CPX (0–20 μM) for 24 h increased the expression of γH2AX, a marker of DNA DSB, in both A549 and A427 cells dose-dependently (Figure 3A,B and Figure S1). In addition, CPX treatment also dose-dependently increased the ATR-mediated phosphorylation of Chk1 on S345 as well as the autophosphorylation of Chk1 on S296, despite dose-dependently reducing the protein level of Chk1 in the cells (Figure 3A,B and Figure S1).

Next, we tested whether PRE strengthens the anticancer effect of CPX on NSCLC cells by bolstering CPX-induced DNA damage. As shown in Figure 3A,B and Figure S1, pretreatment with 20 nM of PRE for 2 h increased the phosphorylation of Chk1 on S345 (mediated by ATR) induced by CPX at low concentrations (2.5 or 5 μM), but completely blocked CPX-induced autophosphorylation of Chk1 (S296) in A549 and A427 cells, indicating the selective inhibition of PRE on Chk1 in the cells. Of interest, pretreatment with PRE profoundly increased the expression of γH2AX induced by CPX, even at low concentrations (2.5–5 μM), as detected by Western blotting (Figure 3A,B and Figure S1).

In addition, our immunofluorescence staining also showed that approximately 5% of A549 cells were γH2AX-positive when treated with vehicle (DMSO), while about 55% and 15% cells were γH2AX-positive when treated with CPX (5 μM) and PRE (20 nM) for 48 h, respectively (Figure 3C,D). The combined treatment significantly increased γH2AX-positive cells to around 74%, compared to each single treatment (Figure 3C,D). Collectively, these observations support the notion that inhibition of Chk1 with PRE enhances the anticancer effect of CPX on NSCLC cells by inducing excess DNA damage in NSCLC cells.

### 3.3. Combined Treatment of CPX and PRE Induces Cell Cycle Arrest in the G1/G0 Phase in NSCLC Cells

**Figure 4:**
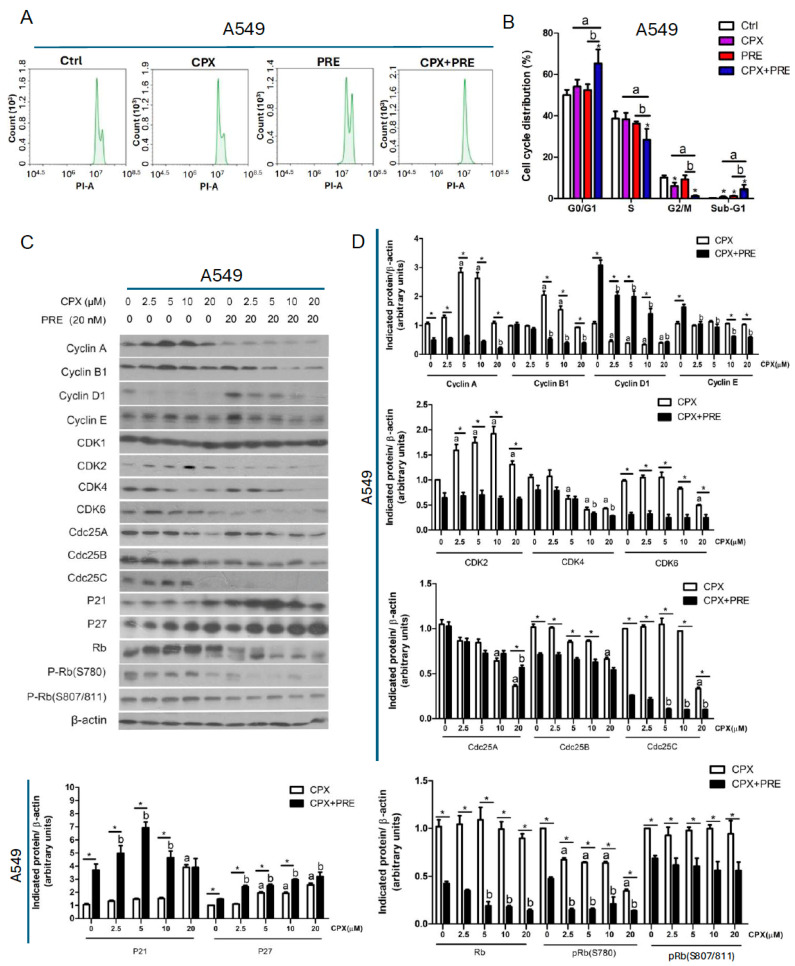
Combined treatment of CPX and PRE induces cell cycle arrest in the G1/G0 phase in NSCLC cells. (**A**–**D**) A549 cells were pretreated with or without prexasertib (20 nM) for 2 h and then exposed to CPX (0–20 μM) for 24 h. (**A**) The cells were harvested, followed by PI staining and flow cytometry. (**B**) Shown is a summary of cell cycle distribution in (**A**). Results are presented as mean ± SD (*n* = 3). * *p* < 0.05, compared to control group; a: *p* < 0.05, CPX treatment group vs. CPX + PRE treatment group; and b: *p* < 0.05, PRE treatment group vs. CPX + PRE treatment group. (**C**) The whole cell lysates were subjected to Western blotting with indicated antibodies. β-actin served as a loading control. Similar results were obtained in at least three independent experiments. (**D**) The protein bands in the Western blots were semi-quantified using NIH ImageJ. Results are presented as mean ± SD (*n* = 3). * *p* < 0.05, CPX treatment group vs. CPX + PRE treatment group; a: *p* < 0.05, Control vs. CPX treatment group; and b: *p* < 0.05, Control vs. PRE treatment group.

To understand how PRE potentiates CPX-induced inhibition of cell proliferation, we performed cell cycle analysis. For this, A549 cells were pretreated with 20 nM of PRE for 2 h, and then exposed to 5 µM of CPX for 24 h, followed by propidium iodide (PI) staining and flow cytometry. We found that CPX or PRE treatment slightly increased the cell population in the G0/G1 phase and sub-G1 phase, and correspondingly decreased the cell population in the S phase or G2/M phase (Figure 4A,B). The combination treatment remarkably decreased the percent cells in the S and G2/M phases and increased the percent cells in the G1/G0 phase, indicating a G1/G0 cell cycle arrest in the cells (Figure 4A,B). Furthermore, we noticed that in response to the combined treatment, the percent cells in the sub-G1 phase were also significantly increased, suggesting that the number of apoptotic cells increased (Figure 4B). Similar results were noticed in A427 cells (Figure S2A,B). Together, our results reveal that the combined treatment with CPX and PRE inhibits NSCLC cell proliferation by arresting the cells in the G1/G0 phase of the cell cycle.

Cyclins and CDKs play a critical role in regulating cell cycle progression [35]. To understand how the combination treatment with CPX and PRE induces the G1/G0 cell cycle arrest in NSCLC cells, we analyzed the expression of CDKs and related regulatory proteins. As shown in Figure 4C,D and Figure S2C,D, the combined treatment of CPX and PRE for 24 h remarkably decreased the cellular protein levels of cyclin A, cyclin B1, CDK2, and CDK6, and increased the levels of the CDK inhibitors p21 (Cip1) and p27 (Kip1) compared to treatment with CPX alone in A549 and A427 cells. CPX treatment dose-dependently reduced the level of CDK4, which was apparently not altered by the addition of PRE. The protein level of CDK1 remained constant regardless of treatment with/without CPX or CPX/PRE in the two cell lines. Notably, treatment with CPX dose-dependently inhibited the expression of cyclin D1, which was strengthened by the addition of PRE in A427 cells and weakened by the addition of PRE in A549 cells.

The phosphatases Cdc25A, Cdc25B and Cdc25C are required for the progression through G1, S and G2/M phases, respectively, by dephosphorylating and activating related CDKs [36]. We found that CPX alone downregulated the expression of Cdc25A and Cdc25B in a dose-dependent manner in A549 and A427 cells. The addition of PRE attenuated high doses of CPX (10 and 20 μM)-induced reduction of Cdc25A, and markedly potentiated CPX-induced reduction of Cdc25B in A549 cells, but it did not significantly alter the levels of Cdc25A and Cdc25B in A427 cells (Figure 4C,D and Figure S2C,D). Of note, the expression of Cdc25C was dose-dependently inhibited by CPX treatment, and almost completely abolished by co-treatment with CPX/PRE in A549 cells, whereas the expression of Cdc25C was not affected by treatment with either CPX or CPX/PRE in A427 cells (Figure 4C,D and Figure S2C,D).

We also noticed that treatment with CPX alone dose-dependently increased the levels of p21 (Cip1) and p27 (Kip1), two CDK inhibitors, in both A549 and A427 cells, which was strengthened by addition of PRE (Figure 4C,D and Figure S2C,D).

Rb is one of the major G1 cyclin/CDK substrates and acts as a crucial check point protein for cell cycle progression from G1 to the S phase [35]. Next, we further investigated the effect of the combined treatment on Rb phosphorylation. Western blotting showed that the CPX treatment increased the protein level of Rb. Also, a lower band, which migrates rapidly and represents the dephosphorylated protein, was observed after 20 μM of CPX treatment and the combined treatment in A549 cells (Figure 4C) as well as the combined treatment in A427 cells (Figure S2C), indicating that the high concentration of CPX or the combined treatment inhibited the phosphorylation of Rb. This was confirmed with the antibodies to phosphorylated Rb (p-Rb, S780; S807/811) (Figure 4C,D and Figure S2C,D). The results indicate that the combined treatment induced accumulation of NSCLC cells in the G0/G1 phase of the cell cycle due to inhibition of Rb, a consequence of inhibition of G1-cyclin/CDKs.

### 3.4. Combined Treatment with CPX and PRE Induces Apoptosis of NSCLC Cells

**Figure 5:**
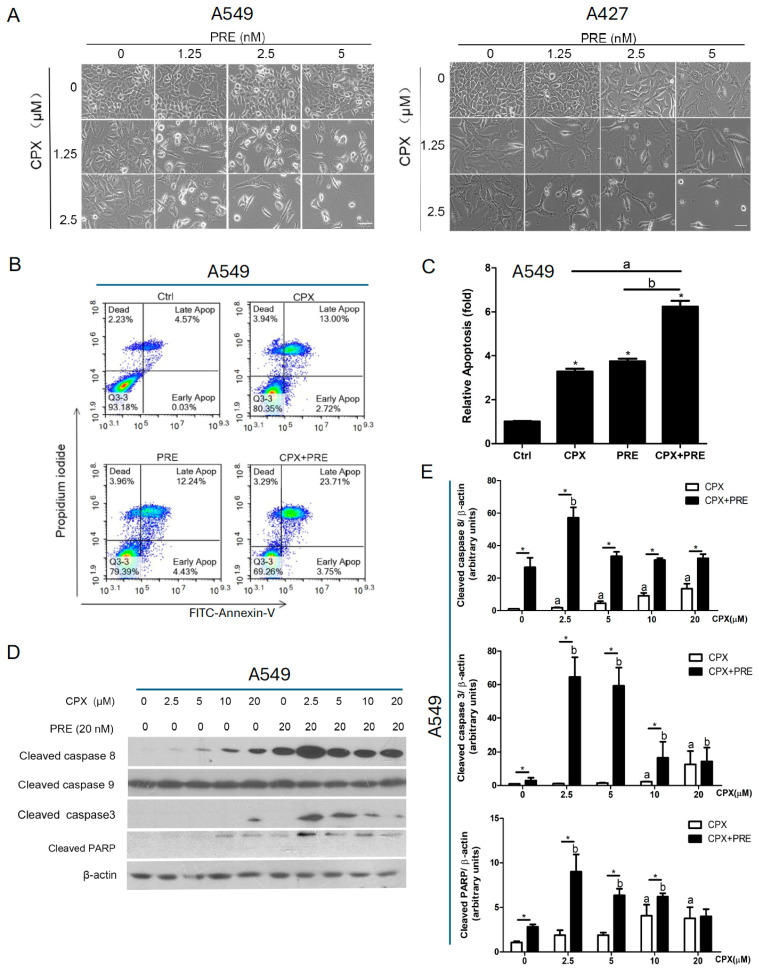
Inhibition of Chk1 with PRE enhances CPX-induced apoptosis of NSCLC cells. (**A**) A549 and A427 cells were treated with CPX (0–2.5 μM) in combination with PRE (0–5 nM) for 5 days. Images were captured with a phase-contrast microscope equipped with the Quick Imaging system. (**B**) A549 cells were treated with CPX (5 μM) or PRE (20 nM) for 72 h. The cells were harvested and stained with Annexin V-FITC and PI, followed by flow cytometry. (**C**) Shown is a summary of the percentage of apoptotic (early + late apoptotic) cells. Results are presented as mean ± SD (*n* = 3). * *p* < 0.05, compared to control group; a: *p* < 0.05, CPX treatment group vs. CPX + PRE treatment group; and b: *p* < 0.05, PRE treatment group vs. CPX + PRE treatment group. (**D**) A549 cells were treated with CPX (0–20 μM) in the presence or absence of PRE (20 nM) for 24 h. The whole cell lysates were subjected to Western blotting with indicated antibodies. β-actin served as a loading control. Similar results were obtained in at least three independent experiments. (**E**) The protein bands in the Western blots were semi-quantified using NIH ImageJ. Results are presented as mean ± SD (*n* = 3). * *p* < 0.05, CPX treatment group vs. CPX + PRE treatment group; a: *p* < 0.05, Control vs. CPX treatment group; and b: *p* < 0.05, Control vs. PRE treatment group.

Mounting evidence has suggested that CPX exerts anticancer activity partly by inducing apoptosis in various cancer cells [7], including lung cancer cells [37,38]. Next, we evaluated whether PRE potentiates the cytotoxicity of CPX by increasing apoptosis in NSCLC cells. For this, we first evaluated the morphology of A549 and A427 cells treated with CPX (0–2.5 μM) in combination with PRE (0–5 nM) for 5 days. We found that the combined treatment more effectively inhibited cell growth relative to the treatment with CPX or PRE alone. Also, in response to the combined treatment, more cells became shrunk or round (Figure 5A), suggesting the induction of apoptosis.

To verify if apoptosis was induced by the combined treatment in A549 cells, we performed Annexin V-FITC and propidium iodide staining for the treated cells, followed by flow cytometry. The lower right and upper right quadrants in the histograms represent the early and late apoptotic cells, respectively. As shown in Figure 5B,C, 72 h treatment of A549 cells with CPX (5 μM) or PRE (20 nM) alone increased the percentage of apoptotic cells by approximately 3.4-fold and 3.6-fold, respectively, and the combined treatment further increased the percentage of apoptosis cells by about 6-fold, compared with the control.

Besides, we noticed that treatment with CPX alone dose-dependently induced the expression of cleaved PARP in both A549 and A427 cells, which was strengthened by the addition of PRE (20 nM) (Figure 5D,E and Figure S3A,B). As the cleavage of PARP is a consequence of activation of the caspase cascade [39], we further examined whether the caspase cascade was activated. Treatment with CPX alone slightly induced the expression of cleaved caspase 3, an effector caspase, which was increased remarkably by the addition of PRE (Figure 5D,E and Figure S3A,B). Of note, the expressions of cleaved caspase 8, an initiator caspase, was induced by CPX alone dose-dependently in A549 and A427 cells, which was strengthened by the addition of PRE (20 nM) (Figure 5D,E and Figure S3A,B). However, cleaved caspase 9, another initiator caspase, was not altered in the cells (Figure 5D,E and Figure S3A,B).

**Figure 6:**
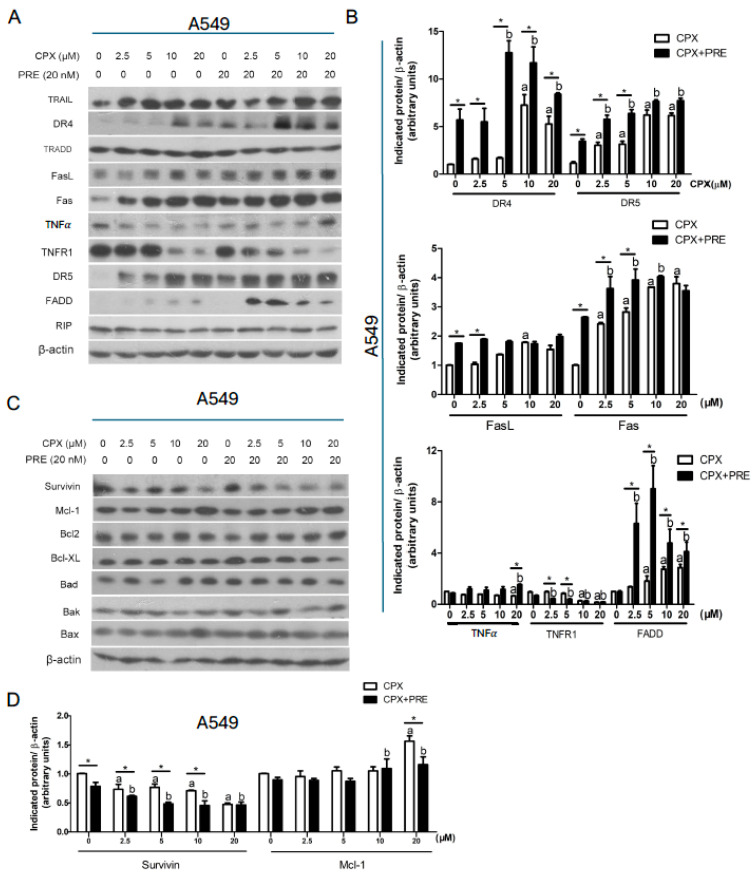
PRE enhances CPX-induced apoptotic cell death through activating both the extrinsic and intrinsic apoptosis pathways in NSCLC cells. (**A**–**D**) A549 cells were treated with CPX (0–20 μM) in the presence or absence of PRE (20 nM) for 24 h. The whole cell lysates were subjected to Western blotting with indicated antibodies (**A**,**C**). β-actin served as a loading control. Similar results were obtained in at least three independent experiments. The protein bands in the Western blots were semi-quantified using NIH ImageJ (**B**,**D**). Results are presented as mean ± S.D. (*n* = 3). * *p* < 0.05, CPX treatment group vs. CPX + PRE treatment group; a: *p* < 0.05, Control vs. CPX treatment group; and b: *p* < 0.05, Control vs. PRE treatment group.

Since caspase 8 activation is linked to activation of the extrinsic pathway [39], we further examined whether CPX/PRE treatments affect the expression of the ligands/death receptors as well as adaptor proteins. For this, A549 and A427 cells were pretreated with or without PRE (20 nM) for 2 h, and then treated with CPX (0–20 μM) for 24 h, followed by Western blotting. We found that treatment with CPX alone upregulated the expression of DR4, DR5, Fas, and FADD, and downregulated the expression of TNFR1 in a dose-dependent manner in A549 and A427 cells, which could be potentiated by pretreatment with PRE (Figure 6A,B and Figure S4A,B). The expression of TNFα was increased by the combined treatment, but the expression of TRADD and RIP in the cells was not altered by treatment with CPX or CPX/PRE (Figure 6A,B and Figure S4A,B). 

To better understand the mechanism by which the combined treatment of CPX and PRE induces apoptosis of tumor cells, the intrinsic pathway was also investigated. To this end, A549 and A427 cells were pretreated with or without PRE (20 nM) for 2 h, and then treated with CPX (0–20 μM) for 24 h. Our Western blot analysis showed that treatment with CPX alone did not obviously alter the expression of anti-apoptotic proteins (Bcl2, Bcl-xL) and pro-apoptotic proteins (BAD, BAK), but dose-dependently decreased the level of anti-apoptotic protein survivin in A549 and A427 cells, and the inhibitory effect of CPX on the survivin expression was strengthened by PRE pretreatment (Figure 6C,D and Figure S4C,D). Of note, treatment with high concentrations of CPX (10 and 20 μM) alone increased the expression of BAX, a pro-apoptotic protein, in A427 cells; co-treatment with PRE and low concentrations of CPX (2.5 and 5 μM) also enhanced the expression of BAX in the cells (Figure S4C,D). In addition, treatment with CPX alone increased the expression of anti-apoptotic protein Mcl-1, which could be attenuated by pretreatment with PRE (Figure 6C,D and Figure S4C,D). Taken together, the results indicate that the combined treatment with CPX and PRE induces caspase-dependent apoptosis in NSCLC through both extrinsic and intrinsic pathways.

## 4. Discussion

Chemotherapy remains a mainstay of lung cancer treatment [4]. The anticancer activity of many chemotherapeutic agents relies on the induction of DNA damage, while the activation of DNA damage response (DDR) pathways can enable cancer cells to survive DNA damage, contributing to chemoresistance [1,25,26,27]. Our previous studies have shown that CPX can inhibit cell proliferation and apoptosis in rhabdomyosarcoma (Rh30) and breast cancer (MDA-MB-231) cells [10]; meanwhile, CPX can induce DNA damage, which activates the ATR-Chk1 DDR pathway [21]. Hence, we postulated that inhibition of Chk1 may potentiate the anticancer activity of CPX. Here, for the first time, we present evidence that CPX dose-dependently inhibits cell proliferation and induces apoptotic cell death in NSCLC cells (A549 and A427), which could be potentiated by the addition of PRE, a Chk1 inhibitor. The findings support our hypothesis.

CPX acts as an anticancer agent by targeting a variety of signaling molecules [7]. It is worth mentioning that CPX is quite cytotoxic to tumor cells rather than normal cells. We have shown that treatment with CPX (2–5–10 μM) dose-dependently reduces cell viability in cancer cells (e.g., A549, A427, and Rh30) cells, but does not exhibit significant cytotoxicity in normal human primary fibroblasts [24]. In the current study, we did not test the cytotoxicity of CPX/PRE combination in normal human primary fibroblasts. However, in our preliminary animal study, we have noticed that treatment with CPX (10 mg/kg, oral gavage once daily) or PRE (10 mg/kg, S.C., twice daily) alone or CPX/PRE combination did not exhibit significant toxicity in the triple-negative breast cancer (MDA-MB-231) xenograft mice model. Therefore, our observations suggest that the combination of CPX/PRE may represent a potential tumor-selective therapeutic approach.

Studies have shown the anticancer potential of CPX in lung cancer cells [37,38]. In addition, the anticancer potential of PRE has also been described in lung cancer [40,41]. However, recently, a phase II clinical trial has demonstrated that PRE does not warrant future development as monotherapy in extensive-stage small-cell lung cancer [42]. In this study, we observed that the combined treatment with CPX and PRE exhibits more potent anticancer activity compared to the single treatment with CPX or PRE in NSCLC. Our in vitro finding provides a basis for further in vivo studies in animal NSCLC models.

It is known that Chk1 can be phosphorylated on S345 by ATR, and autophosphorylated on S296, in response to DNA damage [26,27,28]. In this study, CPX treatment dose-dependently increased p-Chk1 (S345) and p-Chk1 (296) in A549 and A427 cells (Figure 3). This finding suggests that the ATR-Chk1 pathway plays a crucial role in CPX-induced DDR. As expected, pretreatment with the Chk1 inhibitor PRE (20 nM) completely blocked CPX-induced p-Chk1 (296), an autophosphorylation site of Chk1, indicating inhibition of Chk1 (Figure 3 and Figure S1). Interestingly, pretreatment with PRE (20 nM) enhanced p-Chk1 (S345) induced by low concentrations of CPX (2.5–5 μM), but inhibited this phosphorylation induced by high concentrations of CPX (10–20 μM) (Figure 3 and Figure S1). Also, higher levels of γH2AX were observed in the cells following the combined treatments with PRE and low concentrations (2.5–5 μM) of CPX (Figure 3 and Figure S1). This is in line with the cell viability results that PRE was synergistic with CPX when the concentrations were ≤5 μM but was antagonistic when the concentrations were ≥10 μM in the cells (Figure 1C). These observations suggest that PRE should be optimally combined with low doses (2.5–5 μM) of CPX for treatment of NSCLC.

CPX has been shown to induce cell cycle arrest in the G1/G0 phase in many cell lines, including promyeloid leukemia (HL-60) cells [43], Chinese hamster ovary cells [44], human umbilical vein endothelial cells [16], cervical cancer (HeLa) cells [45], neuroblastoma cells [46], rhabdomyosarcoma (Rh30) cells [10], breast cancer (MDA-MB-231) [20], acute lymphoblastic leukemia cells (Jurkat, Molt-4, CEM-C1, CEM-C7) [11], and hepatocellular carcinoma (sk-Hep1 and Huh7) [47]. In this study, we noticed that treatment with CPX alone for 24 h increased the population of NSCLC (A549 and A427) cells in the G1/G0 phase (Figure 4 and Figure S2), which is consistent with the reports of others [36]. In addition, the effect of CPX on cell cycle progression is apparently unrelated to the status of p53. For instance, both normal human umbilical vein endothelial cells (HUVEC) and A549 cells express wild-type p53 [48] while Rh30 cells express mutant p53 [10], but CPX can induce the cell cycle arrest in the G1/G0 phase in HUVEC [16], A549 cells (this study), and Rh30 cells [10].

In this study, we found that treatment with PRE (20 nM) for 24 h slightly but not significantly increased the cell population in the G0/G1 phase and sub-G1 phase, and correspondingly decreased the cell population in the S phase (Figure 4A,B). A study has shown that treatment with PRE (250 nM) for 24 h results in cell cycle arrest in the S phase in breast cancer cells (MDA-MB-231 and MDA-MB-453) [31]. Our results differ from the above finding. Whether the discrepancy is related to the different concentrations of PRE used needs to be further investigated.

In mammalian cells, various cyclin/CDK complexes are formed, which can target appropriate substrates to regulate cell cycle progression [35]. Cyclin D is complexed with CDK4 and/or CDK6 in the early G1 phase, cyclin E and cyclin A are coupled with CDK2 in the late G1-S transition stage and the S phase, respectively, and cyclin B binds to CDK1 in the G2/M phase [35]. In addition, p21 and p27 can bind and inhibit the cyclin D, E and A-dependent kinases [35]. Here, we found that the combined treatment with CPX and PRE inhibited cell proliferation by arresting cells in the G1/G0 phase and reducing cells in the S phase in NSCLC cells. This was partly associated with the downregulation of cyclins (D1, A), CDKs (CDK4, CDK6, CDK2), and upregulation of the CDK inhibitors p21 and p27, resulting in decreased phosphorylation of Rb. Moreover, in response to the combined treatment, the level of CDK1 was not altered, but cyclin B1 was substantially downregulated, which may be linked to the decreased cell population in the G2/M phase.

Cdc25 phosphatases, including Cdc25A, Cdc25B and Cdc25C, and can dephosphorylate and activate G1-, S-, and G2/M-CDK, respectively, thus promoting cell cycle progression [36]. In the current study, in response to the combined treatment with CPX/PRE, the level of Cdc25A was reversed compared to the high dose of CPX (20 μM) treatment alone, but the level of Cdc25B was remarkably reduced in A549 cells. Of note, in response to the combined treatment, the expression of Cdc25C was almost abolished in A549 cells, despite not being altered in A427 cells (Figure 4C,D and Figure S2C,D). It would be interesting to identify the mechanism behind the differential effect of CPX on Cdc25C in A549 and A427 cells. Nevertheless, the above results suggest that the decreased expression of Cdc25A and/or Cdc25B/C may also partly contribute to the enhanced anti-proliferative effect of the CPX/PRE co-treatment.

Apoptosis is mediated by the intrinsic pathway and/or the extrinsic pathway [39]. On the one hand, in the extrinsic pathway, extracellular ligands (e.g., FasL and TNFα) bind to cell surface death receptors (e.g., Fas and TNFR1), which results in activation of initiator caspases (e.g., caspase 8) and finally effector caspases (e.g., caspase 3), leading to apoptosis [39]. On the other hand, in the intrinsic pathway, the imbalance of anti-apoptotic proteins (e.g., Bcl-2, Bcl-xL, Mcl-1, and survivin) and pro-apoptotic proteins (e.g., BAD, BAK, BID, and BAX) results in increased permeability of the mitochondrial membrane and causes the release of cytochrome c, which facilitates the formation of apoptosome, leading to activation of caspase 9 and caspase 3 and finally apoptosis [39]. In this study, we identified that PRE synergized with CPX by inducing apoptotic cell death in NSCLC cells through both the extrinsic and intrinsic apoptosis pathways. This is supported by the findings that co-treatment with CPX and PRE for 24 h increased the protein expression of DR4, DR5, Fas, and FADD (Figure 6A,B and Figure S4A,B), leading to activation of caspase 8, as detected by increased level of cleaved caspase 8 (Figure 5D,E and Figure S3). Additionally, the combined treatment greatly reduced the protein level of the anti-apoptotic protein survivin in both A549 and A427 cell lines (Figure 6C,D and Figure S4C,D). Furthermore, the combined treatment also increased the levels of cleaved caspase 3 and cleaved PARP, two markers of apoptosis, compared to treatment with CPX alone.

## 5. Conclusions

This study has demonstrated that CPX is synergistic with PRE in inhibiting cell proliferation and inducing apoptosis of NSCLC (A549 and A427) cells. The combined treatment with CPX and PRE increases the cell population in the G1/G0 phase, which is associated with downregulation of cyclins D1/A, and CDK4/6/2, and upregulation of the CDK inhibitors p21 and p27. Also, the addition of PRE enhances CPX-induced apoptosis, which is linked to increased expression of DR4, DR5, Fas, and FADD and decreased expression of survivin, resulting in activation of caspases 8/3 as well as cleavage of PARP. Our results support the notion that inhibition of Chk1 with PRE can enhance the anticancer activity of CPX in NSCLC cells. Both CPX and PRE have successfully completed early phase clinical trials as monotherapy in cancer patients. It would be of interest to determine if this combination exhibits promising activity in animal models and eventually in cancer patients.

## Data Availability

The authors confirm that the data supporting the findings of this study are available within the article or its Supplementary Materials.

## Supplementary Materials

The following supporting information can be downloaded at: https://www.mdpi.com/article/10.3390/cells13211752/s1. Figure S1. Inhibition of Chk1 with PRE strengthens CPX-induced DNA damage in A427 cells. (A,B) A427 cells were pretreated with or without prexasertib (20 nM) for 2 h and then exposed to CPX (0–20 mM) for 24 h. The whole cell lysates were subjected to Western blotting with indicated antibodies. β-actin served as a loading control. Similar results were obtained in at least three independent experiments. (B) The protein bands in the Western blots were semi-quantified using NIH ImageJ. Results are presented as mean ± S.D. (*n* = 3). * *p* < 0.05, CPX treatment group vs. CPX + PRE treatment group, a: *p* < 0.05, Control vs. CPX treatment group. b: *p* < 0.05, Control vs. PRE treatment group. Figure S2. Combined treatment of CPX and PRE induces cell cycle arrest in the G1/G0 phase in A427 cells. (A–D) A427 cells were pretreated with or without prexasertib (20 nM) for 2 h and then exposed to CPX (0–20 mM) for 24 h. (A) The cells were harvested, followed by PI staining and flow cytometry. (B) Shown is a summary of cell cycle distribution in (A). Results are presented as mean ± S.D. (*n* = 3). * *p* < 0.05, compared to control group; a: *p* < 0.05, CPX treatment group vs. CPX + PRE treatment group; b: *p* < 0.05, PRE treatment group vs. CPX + PRE treatment group. (C) The whole cell lysates were subjected to Western blotting with indicated antibodies. β-actin served as a loading control. Similar results were obtained in at least three independent experiments. (D) The protein bands in the Western blots were semi-quantified using NIH ImageJ. Results are presented as mean ± S.D. (*n* = 3). * *p* < 0.05, CPX treatment group vs. CPX + PRE treatment group, a: *p* < 0.05, Control vs. CPX treatment group. b: *p* < 0.05, Control vs. PRE treatment group. Figure S3. Inhibition of Chk1 with PRE enhances CPX-induced apoptosis of A427 cells. (A) A427 cells were treated with CPX (0–20 mM) in the presence or absence of PRE (20 nM) for 24 h. The whole cell lysates were subjected to Western blotting with indicated antibodies. β-actin served as a loading control. Similar results were obtained in at least three independent experiments. (B) The protein bands in the Western blots were semi-quantified using NIH ImageJ. Results are presented as mean ± S.D. (*n* = 3). * *p* < 0.05, CPX treatment group vs. CPX + PRE treatment group, a: *p* < 0.05, Control vs. with CPX treatment group. b: *p* < 0.05, Control vs. PRE treatment group. Figure S4. PRE enhances CPX-induced apoptotic cell death by activating both the extrinsic and intrinsic apoptosis pathways in A427 cells. (A–D) A427 cells were treated with CPX (0–20 mM) in the presence or absence of PRE (20 nM) for 24 h. The whole cell lysates were subjected to Western blotting with indicated antibodies (A,C). β-actin served as a loading control. Similar results were obtained in at least three independent experiments. The protein bands in the Western blots were semi-quantified using NIH ImageJ (B,D). Results are presented as mean ± S.D. (*n* = 3). * *p* < 0.05, CPX treatment group vs. CPX + PRE treatment group, a: *p* < 0.05, Control vs. CPX treatment group. b: *p* < 0.05, Control vs. PRE treatment group.

## Abbreviations

ATM, ataxia telangiectasia mutated; ATR, ataxia telangiectasia and rad3-related protein; CPX, ciclopirox olamine; AMPK, AMP-activated protein kinase; Cdc25B/C, cell division cycle 25 B/C; CDK2/4/6, cyclin-dependent kinases 2/4/6; Chk1, checkpoint kinase 1; DAPI, 4′,6-diamidino-2-phenylindole; DMEM, Dulbecco’s Modified Eagle’s Medium; DR4/5, *death* receptors 4/5; DSB, double-strand-breaks; FADD, *FAS*-associated death domain protein; FBS, fetal bovine serum; NSCLC, non-small cell lung cancer; PARP, poly (ADP ribose) polymerase; PBS, phosphate buffered saline; PRE, Prexasertib; ROS, reactive oxygen species; Rb, retinoblastoma protein; Ser, serine; and TNFα, tumor necrosis factor alpha.

## References

[1] Rotow J., Bivona T.G. (2017). Understanding and targeting resistance mechanisms in NSCLC. Nat. Rev. Cancer.

[2] Cooper A.J., Sequist L.V., Lin J.J. (2022). Third-generation EGFR and ALK inhibitors: Mechanisms of resistance and management. Nat. Rev. Clin. Oncol..

[3] Otano I., Ucero A.C., Zugazagoitia J., Paz-Ares L. (2023). At the crossroads of immunotherapy for oncogene-addicted subsets of NSCLC. Nat. Rev. Clin. Oncol..

[4] Hellmann M.D., Li B.T., Chaft J.E., Kris M.G. (2016). Chemotherapy remains an essential element of personalized care for persons with lung cancers. Ann. Oncol..

[5] Gupta A.K. (2001). Ciclopirox: An overview. Int. J. Dermatol..

[6] Subissi A., Monti D., Togni G., Mailland F. (2010). Ciclopirox. Drugs.

[7] Huang Z., Huang S. (2021). Reposition of the Fungicide Ciclopirox for Cancer Treatment. Recent Pat. Anticancer Drug Discov..

[8] Eberhard Y., McDermott S.P., Wang X., Gronda M., Venugopal A., Wood T.E., Hurren R., Datti A., Batey R.A., Wrana J. (2009). Chelation of intracellular iron with the antifungal agent ciclopirox olamine induces cell death in leukemia and myeloma cells. Blood.

[9] Goss K.L., Gordon D.J. (2016). Gene expression signature based screening identifies ribonucleotide reductase as a candidate therapeutic target in Ewing sarcoma. Oncotarget.

[10] Zhou H., Shen T., Luo Y., Liu L., Chen W., Xu B., Han X., Pang J., Rivera C.A., Huang S. (2010). The antitumor activity of the fungicide ciclopirox. Int. J. Cancer.

[11] Wu J., Liu H., Zhang G., Gu L., Zhang Y., Gao J., Wei Y., Ma Z. (2016). Antileukemia Effect of Ciclopirox Olamine Is Mediated by Downregulation of Intracellular Ferritin and Inhibition β-Catenin-c-Myc Signaling Pathway in Glucocorticoid Resistant T-ALL Cell Lines. PLoS ONE.

[12] Yang J., Milasta S., Hu D., AlTahan A.M., Interiano R.B., Zhou J., Davidson J., Low J., Lin W., Bao J. (2017). Targeting Histone Demethylases in MYC-Driven Neuroblastomas with Ciclopirox. Cancer Res..

[13] Zhou J., Zhang L., Wang M., Zhou L., Feng X., Yu L., Lan J., Gao W., Zhang C., Bu Y. (2019). CPX Targeting DJ-1 Triggers ROS-induced Cell Death and Protective Autophagy in Colorectal Cancer. Theranostics.

[14] Kim Y., Schmidt M., Endo T., Lu D., Carson D., Schmidt-Wolf I.G. (2011). Targeting the Wnt/beta-catenin pathway with the antifungal agent ciclopirox olamine in a murine myeloma model. In Vivo.

[15] Schmeel L.C., Schmeel F.C., Kim Y., Endo T., Lu D., Schmidt-Wolf I.G. (2013). Targeting the Wnt/beta-catenin pathway in multiple myeloma. Anticancer Res..

[16] Clement P.M., Hanauske-Abel H.M., Wolff E.C., Kleinman H.K., Park M.H. (2002). The antifungal drug ciclopirox inhibits deoxyhypusine and proline hydroxylation, endothelial cell growth and angiogenesis in vitro. Int. J. Cancer.

[17] Mémin E., Hoque M., Jain M.R., Heller D.S., Li H., Cracchiolo B., Hanauske-Abel H.M., Pe’ery T., Mathews M.B. (2014). Blocking eIF5A modification in cervical cancer cells alters the expression of cancer-related genes and suppresses cell proliferation. Cancer Res..

[18] Fujimura K., Wright T., Strnadel J., Kaushal S., Metildi C., Lowy A.M., Bouvet M., Kelber J.A., Klemke R.L. (2014). A hypusine–eIF5A–PEAK1 switch regulates the pathogenesis of pancreatic cancer. Cancer Res..

[19] Luo Y., Zhou H., Liu L., Shen T., Chen W., Xu B., Han X., Zhang F., Scott R.S., Alexander J.S. (2011). The fungicide ciclopirox inhibits lymphatic endothelial cell tube formation by suppressing VEGFR-3-mediated ERK signaling pathway. Oncogene.

[20] Shen T., Shang C., Zhou H., Luo Y., Barzegar M., Odaka Y., Wu Y., Huang S. (2017). Ciclopirox inhibits cancer cell proliferation by suppression of Cdc25A. Genes Cancer.

[21] Shen T., Zhou H., Shang C., Luo Y., Wu Y., Huang S. (2018). Ciclopirox activates ATR-Chk1 signaling pathway leading to Cdc25A protein degradation. Genes Cancer.

[22] Zhou H., Shang C., Wang M., Shen T., Kong L., Yu C., Ye Z., Luo Y., Liu L., Li Y. (2016). Ciclopirox olamine inhibits mTORC1 signaling by activation of AMPK. Biochem. Pharmacol..

[23] Sen S., Hassane D.C., Corbett C., Becker M.W., Jordan C.T., Guzman M.L. (2013). Novel mTOR inhibitory activity of ciclopirox enhances parthenolide antileukemia activity. Exp. Hematol..

[24] Shang C., Zhou H., Liu W., Shen T., Luo Y., Huang S. (2020). Iron chelation inhibits mTORC1 signaling involving activation of AMPK and REDD1/Bnip3 pathways. Oncogene.

[25] Zhou B.B., Elledge S.J. (2000). The DNA damage response: Putting checkpoints in perspective. Nature.

[26] Abraham R.T. (2001). Cell cycle checkpoint signaling through the ATM and ATR kinases. Genes Dev..

[27] Bartek J., Lukas J. (2003). Chk1 and Chk2 kinases in checkpoint control and cancer. Cancer Cell.

[28] Zhang Y., Hunter T. (2014). Roles of Chk1 in cell biology and cancer therapy. Int. J. Cancer.

[29] Angius G., Tomao S., Stati V., Vici P., Bianco V., Tomao F. (2020). Prexasertib, a checkpoint kinase inhibitor: From preclinical data to clinical development. Cancer Chemother. Pharmacol..

[30] Lowery C.D., VanWye A.B., Dowless M., Blosser W., Falcon B.L., Stewart J., Stephens J., Beckmann R.P., Lin A.B., Stancato L.F. (2017). The Checkpoint Kinase 1 Inhibitor Prexasertib Induces Regression of Preclinical Models of Human Neuroblastoma. Clin. Cancer Res..

[31] Mani C., Jonnalagadda S., Lingareddy J., Awasthi S., Gmeiner W.H., Palle K. (2019). Prexasertib treatment induces homologous recombination deficiency and synergizes with olaparib in triple-negative breast cancer cells. Breast Cancer Res..

[32] Heidler C.L., Roth E.K., Thiemann M., Blattmann C., Perez R.L., Huber P.E., Kovac M., Amthor B., Neu-Yilik G., Kulozik A.E. (2020). Prexasertib (LY2606368) reduces clonogenic survival by inducing apoptosis in primary patient-derived osteosarcoma cells and synergizes with cisplatin and talazoparib. Int. J. Cancer.

[33] Do K.T., Kochupurakkal B., Kelland S., de Jonge A., Hedglin J., Powers A., Quinn N., Gannon C., Vuong L., Parmar K. (2021). Phase 1 Combination Study of the CHK1 Inhibitor Prexasertib and the PARP Inhibitor Olaparib in High-grade Serous Ovarian Cancer and Other Solid Tumors. Clin. Cancer Res..

[34] Chou T.C., Talalay P. (1984). Quantitative analysis of dose-effect relationships: The combined effects of multiple drugs or enzyme inhibitors. Adv. Enzym. Regul..

[35] Matthews H.K., Bertoli C., de Bruin R.A.M. (2022). Cell cycle control in cancer. Nat. Rev. Mol. Cell Biol..

[36] Boutros R., Lobjois V., Ducommun B. (2007). CDC25 phosphatases in cancer cells: Key players? Good targets?. Nat. Rev. Cancer.

[37] Lu J., Li Y., Gong S., Wang J., Lu X., Jin Q., Lu B., Chen Q. (2022). Ciclopirox targets cellular bioenergetics and activates ER stress to induce apoptosis in non-small cell lung cancer cells. Cell Commun. Signal..

[38] Yin J., Che G., Jiang K., Zhou Z., Wu L., Xu M., Liu J., Yan S. (2022). Ciclopirox Olamine Exerts Tumor-Suppressor Effects *via* Topoisomerase II Alpha in Lung Adenocarcinoma. Front. Oncol..

[39] Yuan J., Ofengeim D. (2024). A guide to cell death pathways. Nat. Rev. Mol. Cell Biol..

[40] Sen T., Tong P., Stewart C.A., Cristea S., Valliani A., Shames D.S., Redwood A.B., Fan Y.H., Li L., Glisson B.S. (2017). CHK1 Inhibition in Small-Cell Lung Cancer Produces Single-Agent Activity in Biomarker-Defined Disease Subsets and Combination Activity with Cisplatin or Olaparib. Cancer Res..

[41] Hsu W.H., Zhao X., Zhu J., Kim I.K., Rao G., McCutcheon J., Hsu S.T., Teicher B., Kallakury B., Dowlati A. (2019). Checkpoint Kinase 1 Inhibition Enhances Cisplatin Cytotoxicity and Overcomes Cisplatin Resistance in SCLC by Promoting Mitotic Cell Death. J. Thorac. Oncol..

[42] Byers L.A., Navarro A., Schaefer E., Johnson M., Özgüroğlu M., Han J.Y., Bondarenko I., Cicin I., Dragnev K.H., Abel A. (2021). A Phase II Trial of Prexasertib (LY2606368) in Patients With Extensive-Stage Small-Cell Lung Cancer. Clin. Lung Cancer.

[43] Hoffman B.D., Hanauske-Abel H.M., Flint A., Lalande M. (1991). A new class of reversible cell cycle inhibitors. Cytometry.

[44] Levenson V., Hamlin J.L. (1993). A general protocol for evaluating the specific effects of DNA replication inhibitors. Nucleic Acids Res..

[45] Szüts D., Krude T. (2004). Cell cycle arrest at the initiation step of human chromosomal DNA replication causes DNA damage. J. Cell Sci..

[46] Sidarovich V., Adami V., Gatto P., Greco V., Tebaldi T., Tonini G.P., Quattrone A. (2015). Translational downregulation of HSP90 expression by iron chelators in neuroblastoma cells. Mol. Pharmacol..

[47] Wan X., Xiang J., Fan H., Jiang Y., Lu Y., Zhang C., Zhang Y., Chen Q., Lei Y. (2023). Ciclopirox Olamine Induces Proliferation Inhibition and Protective Autophagy in Hepatocellular Carcinoma. Pharmaceuticals.

[48] Sun Q., Guo Y., Liu X., Czauderna F., Carr M.I., Zenke F.T., Blaukat A., Vassilev L.T. (2019). Therapeutic Implications of p53 Status on Cancer Cell Fate Following Exposure to Ionizing Radiation and the DNA-PK Inhibitor M3814. Mol. Cancer Res..

