# Supplementary material for: Inhibition of Chk1 with Prexasertib Enhances the Anticancer Activity of Ciclopirox in Non-Small Cell Lung Cancer Cells
Source: Cells. 2024 Oct 23;13(21):1752. doi: 10.3390/cells13211752 (PMC11544771; doi:10.3390/cells13211752)
Supplement: Supplementary file 1 [file cells-13-01752-s001.zip › cells-3159293-supplementary.pdf]

cells

MDPI

Supplementary Material

Inhibition of Chk1 with Prexasertib Enhances the Anticancer Activity of Ciclopirox in Non-Small Cell Lung Cancer Cells

Zhu Huang[1,2], Wenjing Li[2], Yan Wu[2], Bing Cheng[1], Shile Huang[3,4,5,*]

1 Department of Biochemistry and Molecular Biology, Louisiana State University Health Sciences Center, 1501 Kings Highway, Shreveport, LA 71130-3932, USA
2 Collaborative Innovation Center of Targeted Development of Medicinal Resources, College of Life Science, Anqing Normal University, Anqing, Anhui, 246011, China
3 Department of Hematology and Oncology, Louisiana State University Health Sciences Center, 1501 Kings Highway, Shreveport, LA 71130-3932, USA;
4 Feist-Weiller Cancer Center, Louisiana State University Health Sciences Center, Shreveport, LA 71130-3932, USA
* Correspondence: shile.huang@lsuhs.edu (S.H.)

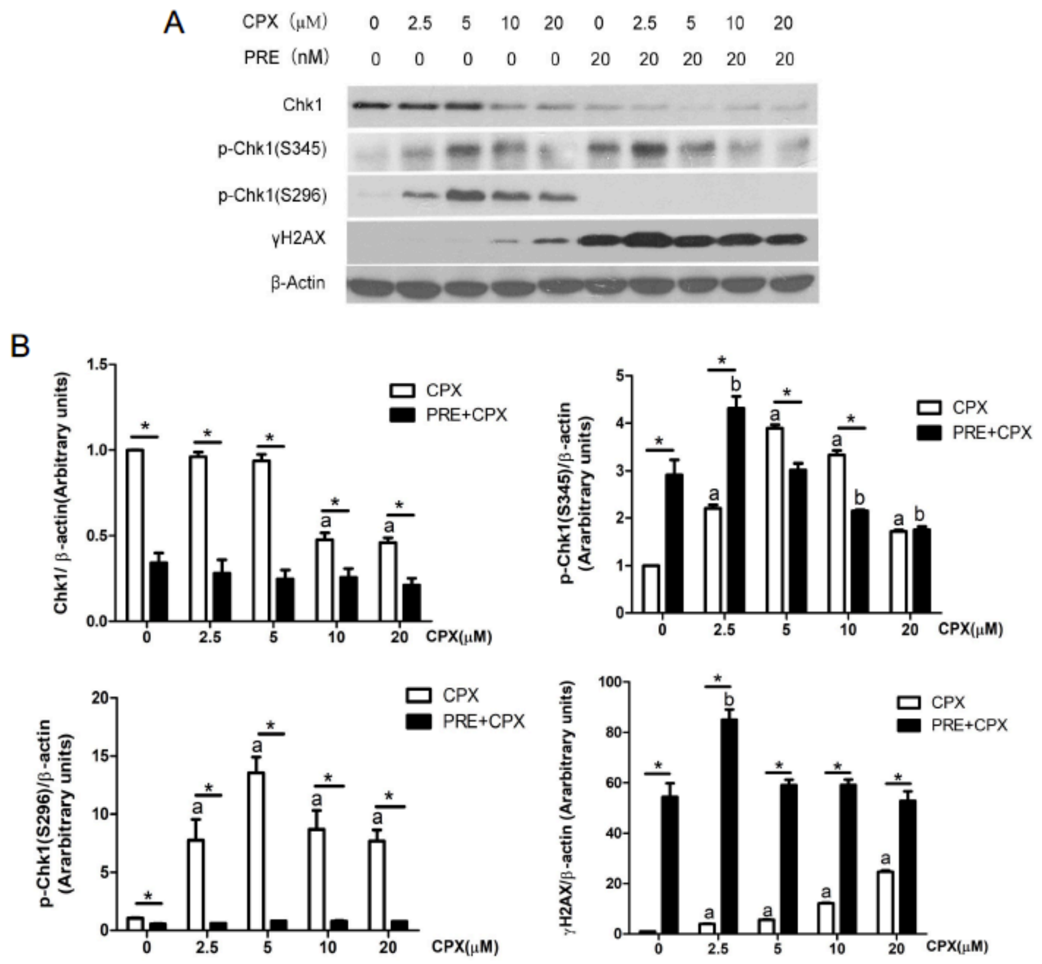

Figure S1. Inhibition of Chk1 with PRE strengthens CPX-induced DNA damage in A427 cells. (A–B) A427 cells were pretreated with or without prexasertib (20 nM) for 2 h and then exposed to CPX (0–20 μM) for 24 h. The whole cell lysates were subjected to Western blotting with indicated antibodies. β-actin served as a loading control. Similar results were obtained in at least three independent experiments. (B) The protein bands in the Western blots were semi-quantified using NIH ImageJ. Results are presented as mean ± S.D. (n = 3). *$P < 0.05$, CPX treatment group vs CPX+PRE treatment group, a: $P < 0.05$, Control vs CPX treatment group. b: $P < 0.05$, Control vs PRE treatment group.

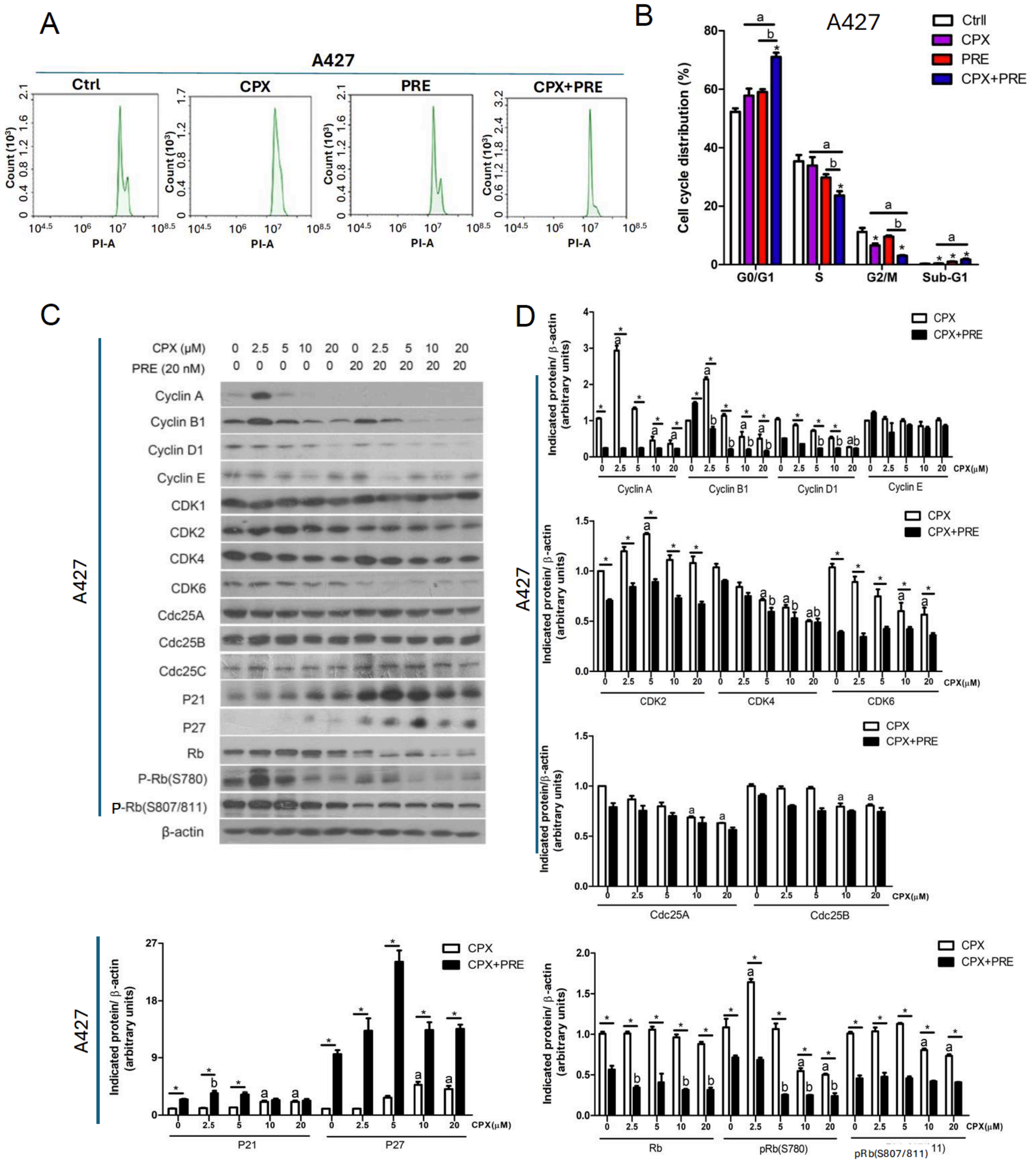

Figure S2. Combined treatment of CPX and PRE induces cell cycle arrest in the G1/G0 phase in A427 cells. (A–D) A427 cells were pretreated with or without prexasertib (20 nM) for 2 h and then exposed to CPX (0–20 μM) for 24 h. (A) The cells were harvested, followed by PI staining and flow cytometry. (B) Shown is a summary of cell cycle distribution in (A). Results are presented as mean ± S.D. (n = 3). **P* < 0.05, compared to control group; a: *P* < 0.05, CPX treatment group vs CPX+PRE treatment group; b: *P* < 0.05, PRE treatment group vs CPX+PRE treatment group. (C) The whole cell lysates were subjected to Western blotting with indicated antibodies. β-actin served as a loading control. Similar results were obtained in at least three independent experiments. (D) The protein bands in the Western blots were semi-quantified using NIH ImageJ. Results are presented as mean ± S.D. (n = 3). **P* < 0.05, CPX treatment group vs CPX+PRE treatment group, a: *P* < 0.05, Control vs CPX treatment group. b: *P* < 0.05, Control vs PRE treatment group.

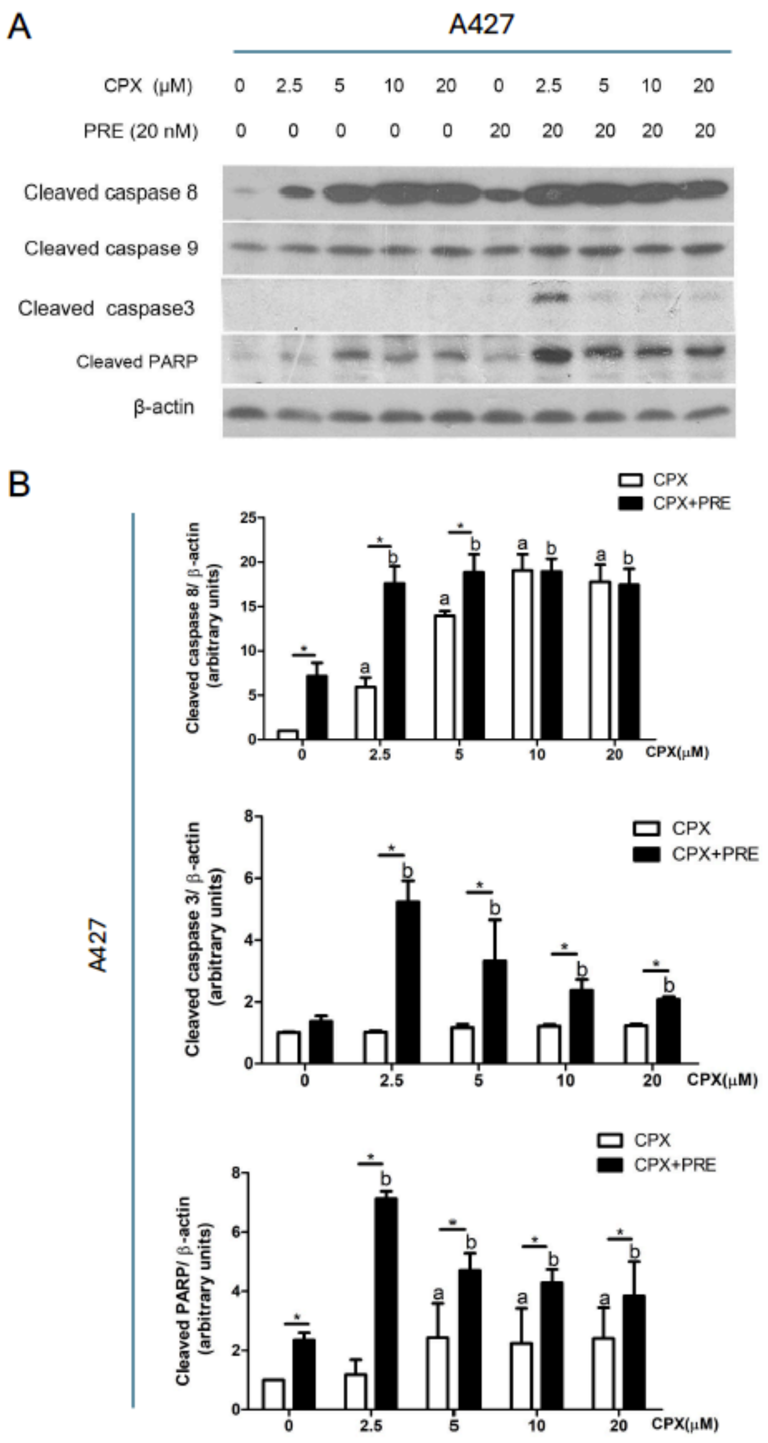

Figure S3. Inhibition of Chk1 with PRE enhances CPX-induced apoptosis of A427 cells. (A) A427 cells were treated with CPX (0–20 μM) in the presence or absence of PRE (20 nM) for 24 h. The whole cell lysates were subjected to Western blotting with indicated antibodies. β-actin served as a loading control. Similar results were obtained in at least three independent experiments. (B) The protein bands in the Western blots were semi-quantified using NIH ImageJ. Results are presented as mean ± S.D. (n = 3). *$P < 0.05$, CPX treatment group vs CPX+PRE treatment group, a: $P < 0.05$, Control vs with CPX treatment group. b: $P < 0.05$, Control vs PRE treatment group.

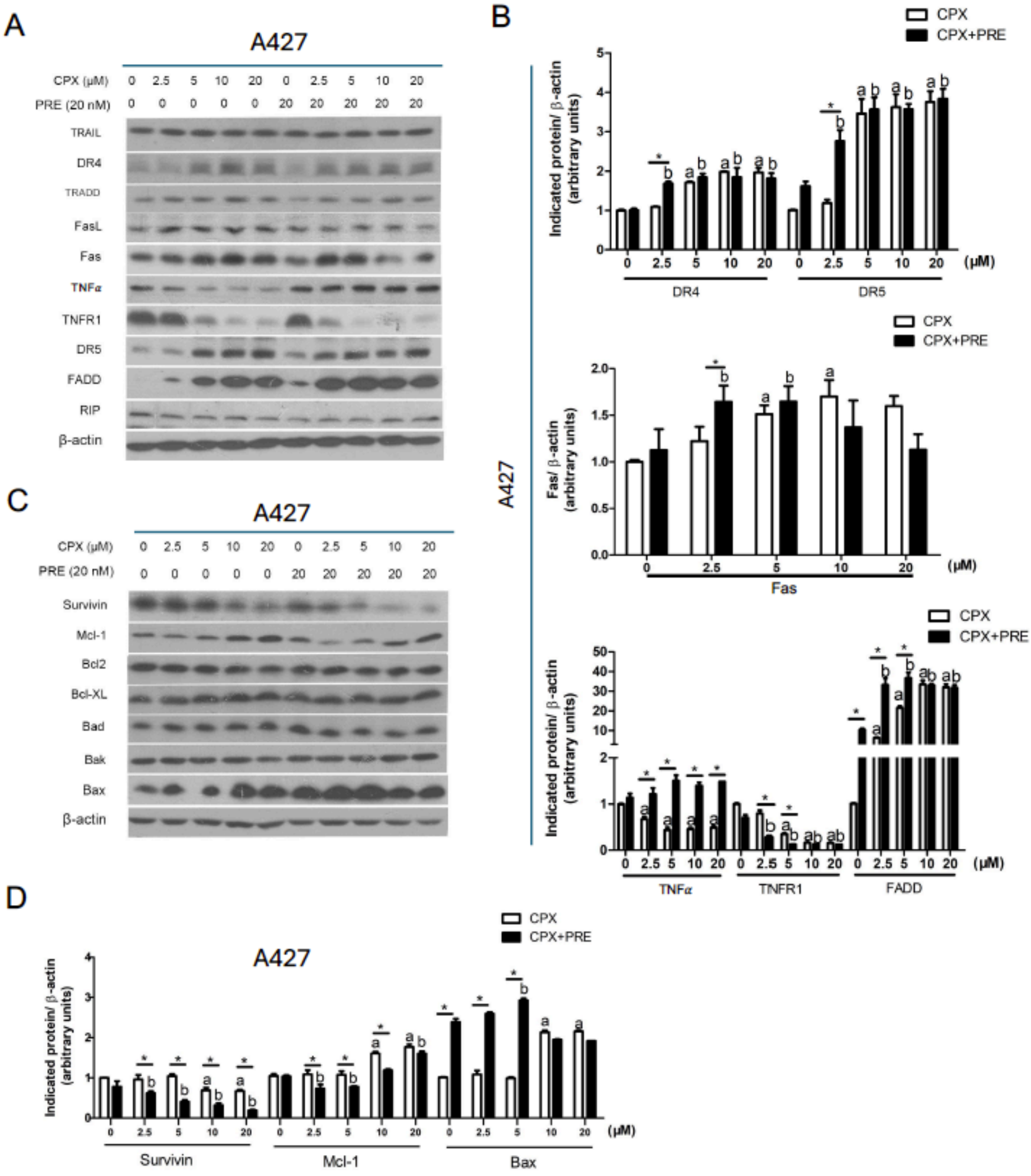

Figure S4. PRE enhances CPX-induced apoptotic cell death by activating both the extrinsic and intrinsic apoptosis pathways in A427 cells. (A–D) A427 cells were treated with CPX (0–20 μM) in the presence or absence of PRE (20 nM) for 24 h. The whole cell lysates were subjected to Western blotting with indicated antibodies (A, C). β-actin served as a loading control. Similar results were obtained in at least three independent experiments. The protein bands in the Western blots were semi-quantified using NIH ImageJ (B, D). Results are presented as mean ± S.D. (n = 3). **P* < 0.05, CPX treatment group vs CPX+PRE treatment group, a: *P* < 0.05, Control vs CPX treatment group. b: *P* < 0.05, Control vs PRE treatment group.
